# Multi-modal remote sensory learning for multi-objects over autonomous devices

**DOI:** 10.3389/fbioe.2025.1430222

**Published:** 2025-05-20

**Authors:** Aysha Naseer, Naif Almudawi, Hanan Aljuaid, Abdulwahab Alazeb, Yahay AlQahtani, Asaad Algarni, Ahmad Jalal, Hui Liu

**Affiliations:** 1 Department of Computer Science, Air University, Islamabad, Pakistan; 2 Department of Computer Science, College of Computer Science and Information System, Najran University, Najran, Saudi Arabia; 3 Computer Sciences Department, College of Computer and Information Sciences, Princess Nourah bint Abdulrahman University (PNU), Riyadh, Saudi Arabia; 4 Department of Informatics and Computer Systems, King Khalid University, Abha, Saudi Arabia; 5 Department of Computer Sciences, Faculty of Computing and Information Technology, Northern Border University, Rafha, Saudi Arabia; 6 Department of Computer Science and Engineering, College of Informatics, Korea University, Seoul, South Korea; 7 Guodian Nanjing Automation Co., Ltd., Nanjing, China; 8 Jiangsu Key Laboratory of Intelligent Medical Image Computing, School of Future Technology, Nanjing University of Information Science and technology, Nanjing, China; 9 Cognitive Systems Lab, University of Bremen, Bremen, Germany

**Keywords:** multi-modal, remote sensing, multi-objects, autonomous devices deep learning, computer vision, posterior probability, likelihood estimation, scene analysis

## Abstract

**Introduction:**

There has been an increasing focus on object segmentation within remote sensing images in recent years due to advancements in remote sensing technology and the growing significance of these images in both military and civilian realms. In these situations, it is critical to accurately and quickly identify a wide variety of objects. In many computer vision applications, scene recognition in aerial-based remote sensing imagery presents a common issue.

**Method:**

However, several challenging elements make this work especially difficult: (i) Different objects have different pixel densities; (ii) objects are not evenly distributed in remote sensing images; (iii) objects can appear differently depending on viewing angle and lighting conditions; and (iv) there are fluctuations in the number of objects, even the same type, in remote sensing images. Using a synergistic combination of Markov Random Field (MRF) for accurate labeling and Alex Net model for robust scene recognition, this work presents a novel method for the identification of remote sensing objects. During the labeling step, the use of MRF guarantees precise spatial contextual modeling, which improves comprehension of intricate interactions between nearby aerial objects. By simultaneously using deep learning model, the incorporation of Alex Net in the following classification phase enhances the model’s capacity to identify complex patterns in aerial images and adapt to a variety of object attributes.

**Results:**

Experiments show that our method performs better than others in terms of classification accuracy and generalization, indicating its efficacy analysis on benchmark datasets such as UC Merced Land Use and AID.

**Discussion:**

Several performance measures were calculated to assess the efficacy of the suggested technique, including accuracy, precision, recall, error, and F1-Score. The assessment findings show a remarkable recognition rate of around 97.90% and 98.90%, on the AID and the UC Merced Land datasets, respectively.

## 1 Introduction

The evolution during the last several years of remote sensing (RS) technologies, in terms of platforms, sensors, and information support, has led to the significant increase in availability of EO data for geospatial analysis. Semantic segmentation is a key task in RS that has implications for a diverse area of applications, such as land use classification (Ahmad et al., 2020; Alazeb et al., 2023), changes detection (Islam et al., 2016; Saha et al., 2019), and environment surveillance (Srivastava et al., 2012; Jalal et al., 2021). However, these fine segmentation algorithms are not practically achievable due to the unavailability of large-scale labeled data and more critically, the quality of the labeled data is poor due to coverage restrictions of the satellite sensors particularly over different geographical terrains and various types of land. Conventionally every pixel in satellite imagery corresponds to a wide geographical region (Naseer et al., 2024a); therefore, pixel-level annotation is not only time consuming but also financially expensive and requires high levels of specialization (Manfreda et al., 2018; Khan et al., 2020).

To this end, there is a trend among scholars trying to employ semi-supervised methods which can minimize the amount of pixel-wise labeling, while using the gray images to extract information (Guo et al., 2020; Liu et al., 2017). Nevertheless, prevalent techniques entail biases in the procedure where the labeled data is selected at random, which in turn results in the massive formation of skewed models and innate inferior performance (Han et al., 2017; Muhammad et al., 2018). Second, segmentation in the remote sensing has problems that are closely related to characteristics of images such as different scales of the objects are caused shooting angles, inequality of counts of various objects, a large number of small objects in the aerial images and etc. Problems of truncation and occlusion make recognition even more challenging (Martin, 2011). Developing on previous attempts at employing remote sensing, computational methods from the past relied on statistical and rule-based methodologies, for instance, the decision tree algorithms or unsupervised clustering; modern deep learning techniques are scalable and more vigorous.

Earlier, classification based on remote sensing data used simple statistical and rule based techniques including decision tree algorithms and unsupervised clustering methods to classify land cover and land use. For example, the research in (Jayanetti et al., 2017) showed how land cover data can be combined with vGI such as Foursquare to land use. However, such methods have limitations associated with requiring the definition of certain thresholds and assumptions which reduces accuracy for complex terrain. Taking inspiration from these methods, deep learning-based methods have just lately been developed as superior and more scalable ways to handle remote sensing jobs. For instance, a model for categorizing suitable land for agriculture based on geographic mapping using deep learning was proposed by (Meedeniya et al., 2020), Deep Learning for Sustainable Agriculture, which greatly increased the forecast accuracy for paddy fields. In the same way, U-net and Fully Convolutional Networks (FCN) were used by (Mahakalanda et al., 2022), Deep learning-based prediction for rubber plantations, to identify the land use of rubber plantations. This process took very little time and achieved a very high accuracy of 94.13%, increasing its applicability in crop monitoring.

The study tackles a number of significant research issues in the fields of image segmentation and remote sensing, such as:

- How can object segmentation in remote sensing images be improved to enhance accuracy and efficiency in identifying multiple objects?
- What role do contextual and spectral-spatial features play in improving scene classification?
- How can AlexNet, when integrated with effective segmentation methods, improve the recognition of complex scenes in remote sensing imagery?
- What is the comparative performance of the proposed hybrid system against existing state-of-the-art methods on benchmark datasets?

In this article, the authors introduce an approach for dealing with the key issues in remote sensing scene recognition. While having several limitations, our approach mitigates those issues as follows: First, it minimizes interference and retains crucial aspects of appearance; second, it addresses the noise aspect and relevant spectral—spatial dependencies. Key contributions of this work include:

- Robust segmentation methods: A time comparison of MRF, FCM, and DBSCAN clustering with MRF as the best performer within the time-space complexity.
- Feature integration for scene recognition: Application of both spectral and spatial information along with the Haralick texture higher-order statistical measures in order to improve the segmentation coherency and correctness.
- Deep learning integration: Use of AlexNet for making use of segmented data in order to enhance the kind of recognition of the complicated scenes with much more details and precision.
- Comprehensive evaluation: Comparison with other methods to recognize the proposed hybrid system using newly established benchmarks AID or UCM, with accuracy rates of 97.90% and 98.90% respectively.

The subsections that follow in this paper follow this format. In Section 2, the body of extant literature is examined in detail. A detailed description of our suggested approach, including segmentation, labeling, feature extraction, and their combination, is provided in Section 3. In Section 4, a thorough analysis of the datasets used, the experimental design used, and the resulting results are discussed. Finally, Section 5 presents the results of the research we did.

## 2 Literature review

We reviewed the literature in a variety of disciplines, including object classification, segmentation, labeling, and scene classification, in order to analyze the complexity of aerial and remote sensing images. This helped us create the right dynamics and metrics for our strategy.

### 2.1 Multi-object segmentation

In remote sensing image processing, segmentation is a crucial activity that attempts to divide an image into similar regions. Semantic segmentation specifically aims to allocate every homogenous region to a unique geographical object category, such as cities, farms, or woods. Semantic segmentation has been approached through a variety of approaches in recent decades, including Markov random field (MRF) models (Zhang et al., 2018). Level sets (Ball and Bruce et al., 2007). Clustering (Ma and Yang, 2009; Liu et al., 2015) and deep learning. Traditionally, early techniques such as clustering anticipated that pixels between distinct objects would display distinguishing traits, whereas pixels representing the same object would have identical characteristics. This method works well for low-to medium-resolution images (Zhao et al., 2019), but is ineffective for high-resolution (HSR) remote sensing images. Within an object in a HSR image, individual pixels may have distinct looks, but certain pixels from different objects may have identical (Längkvist et al., 2016) properties. Optimizing a multi-kernel method designed for semantic segmentation in high spatial resolution remote sensing imagery using an advanced Markov Random Field model effectively improves the robust classification of resilient objects in remote sensing images (He, 2024) in his work proposes the Grouping Prompt Tuning Framework (GoPT) based on semantic grouping for multi-modal image segmentation. This results in the original few-shot learning method with only one percent trainable parameters, and each new prompt tuner method brings state-of-the-art performance across multiple multi-modal segmentation tasks. This work reveals the possibility that efficient training of the foundation models implements early learning to address the multi-modal perception issues of weak transfer and scarce labeled data. To address domain gaps in remotely sensed segmentation tasks, authors (Wang et al., 2024) this work introduces Dynamic Loss Correction (DLC), a novel approach. Therefore, in order to apply machines to cross-domain scenarios, DLC adaptively adjusts loss functions to help establish the correspondence between related feature distributions across domains. This method also improves the model precision in terms of segments by stabilizing ephemeral alignment between features of different sources of data thus recommended for use where data of different types used.

### 2.2 Multi-object recognition

There are several challenges in the field of object classification for researchers. These difficulties include issues like localizing objects (Jain and Anto, 2022; Wang et al., 2022), recognizing and analyzing object connections (Sumbul et al., 2019) recovering hidden features, and classifying objects to produce desired outcomes. The widely accepted bag-of-words (Wang et al., 2017) technique has been the prevalent and effective framework for the classifying and recognizing (Naseer et al., 2024b) of objects in modern times. The bag-of-features approach has been the subject of several remarkable investigations (Ghabrani et al., 2023) present a new approach to classify land cover using spatial information derived from statistical properties of complicated CP and QP SAR data. They use super pixels to represent local spatial relationships and a built graph to express global dependencies. In order to estimate the land cover categorization image, labels are propagated from labeled to unlabeled super-pixels.

Furthermore, in a different study (Ahmed and Jalal et al., 2024), presented a unique illustration method designed for certain object classes. The characteristics of each image category were initially defined by a Gaussian mixture model (GMM). They constructed representations for comparison using the Euclidean distances between the pictures and these GMM models. Class-specific characteristics and visual components might be used to convey an image owing to the concatenation of these representations across all classes. A useful method for using multi-object categorization to identify indoor-outdoor situations is described (Ahmed and Jalal et al., 2024). Entails the process using two different ways to segment imagery, after which multiple kernel learning (MKL) will be employed to classify objects. To improve the classification, this procedure combines area-specific signatures with local descriptors. An approach was presented by (Ansith and Bini, 2022) classified land usage in high-resolution remote sensing images using a modified GAN architecture based on an encoder. The suggested technique feeds a latent vector into the generator after it has been generated by an encoder. Images from high-resolution remote sensing datasets are fed into the encoder. Support Vector Machines (SVM) are recommended by (Sangeetham and Sanam, 2023) as a method for identifying high-resolution images. Principal Component Analysis (PCA) is used to extract features from the pictures prior to classification. Support vector machines are then used to classify the feature vectors that are produced. Detecting an object’s contours and motion. However, there is more possibility for incorporating various remote sensing visualization approaches into image processing because to the present high spatial resolution of remote sensing (RS) images and the decreased difference between RS and natural images (He et al., 2024) in their work, Orientation-Aware Multi-Modal Learning for Road Intersection Identification and Mapping, is centered on the association of orientation-learning to map road intersections using multi-modal data such as LiDAR and imagery. This framework merges spatial and geometric orientation characteristics in the fusion of multi-modal data in order to increase the accuracy of the maps. These methods highlight the need to develop frameworks which directly incorporate modality-specific spatial and geometric characteristics to enhance real-world application performance.

## 3 Materials and methods

### 3.1 System methodology

**FIGURE 1:**
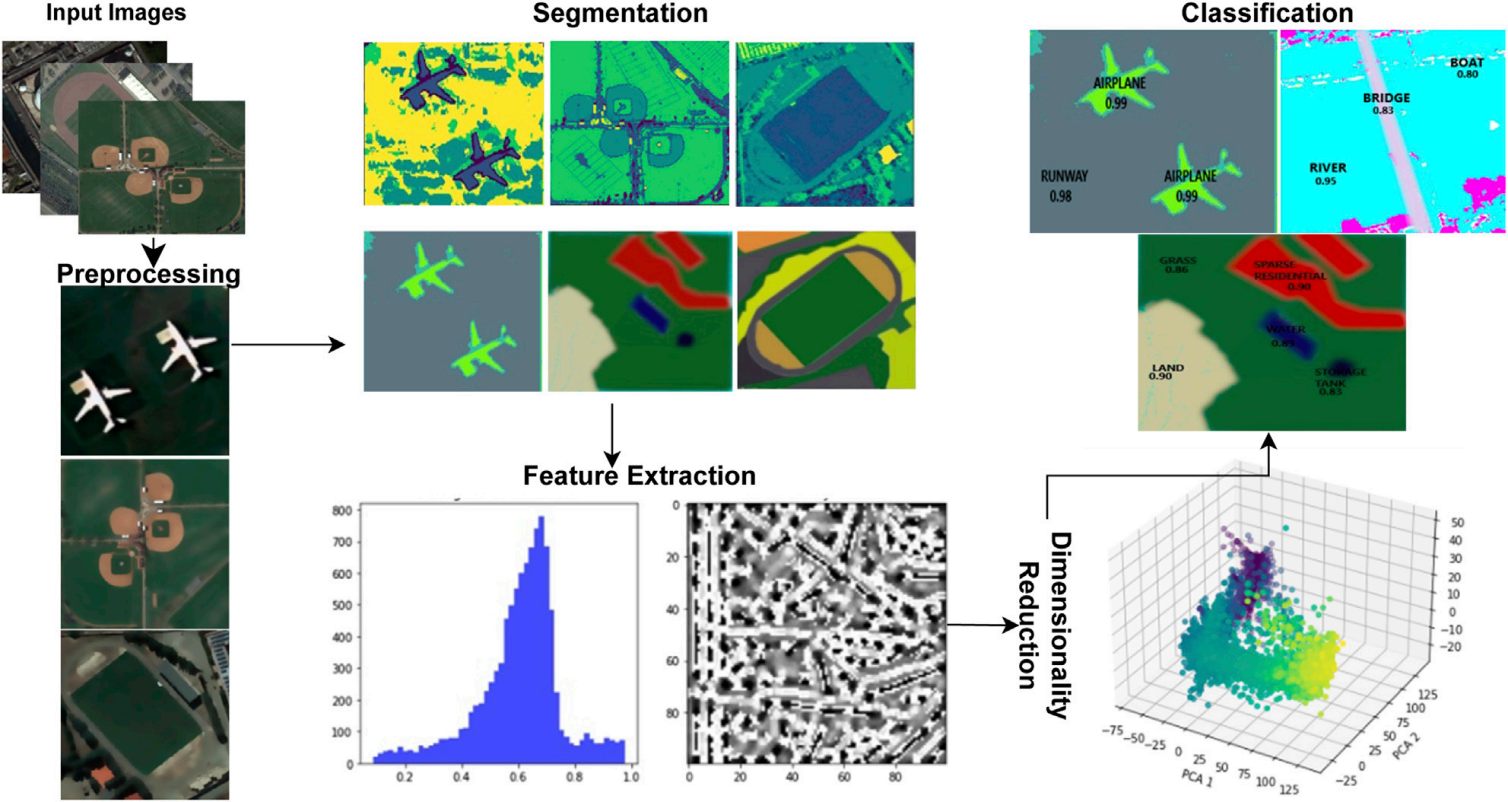
The hierarchical view of the suggested model over remote sensory images.

The basic process of this model begins with the identification and classification of objects seen in images obtained via remote sensing. For image segmentation, it makes use of techniques like FCM, MRF, and DBSCAN clustering. The advantages of MRF in terms of timing efficiency and segmentation accuracy led our team to select it. Our dedication to optimizing processing efficiency while guaranteeing precise object identification in remote sensing imagery is shown in this choice. Using feature extraction, properties including texture, spectral features, and SSF are extracted from the labeled objects. The model can produce more reliable and accurate scene recognition results by fusing AlexNet’s feature learning skills with the contextual information from MRF. In order to offer a graphic depiction of our system’s complex hierarchical structure, Figure 1 describes the hierarchical perspective that encompasses the complex elements and features of our OSC model.

### 3.2 Noise removal

**FIGURE 2:**
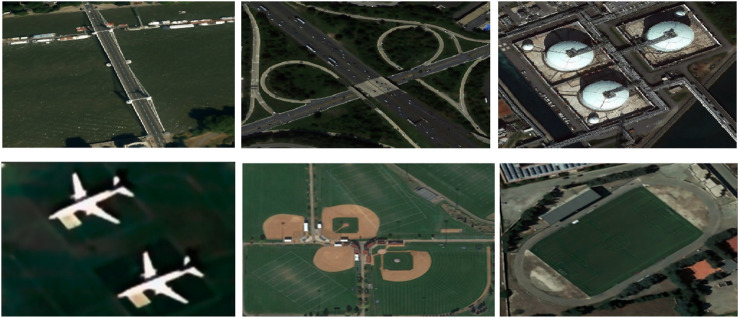
Outcomes of filtered images using Bilateral filter over some images of both datasets.

The bilateral filter is a well-liked image processing method that may be used for a variety of operations, such as edge preservation, noise reduction and smoothing. Whtaen pursuing filtering, the bilateral filter (Tripathi and Mukhopadhay, 2012) considers both the spatial distance and the intensity difference between pixels. This dual-domain method enables it to blur an image while maintaining crucial edges and precise details. The weights in the weighted average of neighboring pixels computed by the bilateral filter rely on both the spatial and intensity distances. The weighted average for each pixel may be written mathematically (Hu et al., 2023) as given in Equations 1-3:

$$I\left(x,y\right)=\frac{1}{\;W\left(x,y\right)}\sum_{p\in N\left(x,y\right)}^{1}I\left(p\right).G_{s}\left(\left\|p-\left(x,y\right)\right\|\right)G_{r}\left(\left\|I\left(p\right)-I\left(x,y\right)\right\|\right)$$ (1)

where I (x, y) is the intensity value of the pixel being filtered, the normalization factor W (x, y) makes sure the total weight adds up to 1. N (x, y) represents neighboring pixels around (x, y).

$$G_{s}\left\|p-\left(x,y\right)\right\|=\exp\left(-\frac{{\left\|p-\left(x,y\right)\right\|}^{2}}{2\delta_{s}^{2}}\right)$$ (2)

$$G_{r}\left\|I\left(p\right)-I\left(x,y\right)\right\|=\exp\left(-\frac{{\left\|I\left(p\right)-I\left(x,y\right)\right\|}^{2}}{2\delta_{r}^{2}}\right)$$ (3)

where “p” represents the neighboring pixel location, *δ*_*s*_ spatial standard deviation, and *δ*_*r*_ range standard deviation. The filtered result is shown in Figure 2.

### 3.3 Semantic segmentation

In order to simplify image representation for analysis, segmentation is dividing an image into homogenous and significant parts. The goal is to produce regions with comparable visual features, such as color or texture. In contrast, labeling reveals the meaning or class of each segment by assigning semantic labels to each one that was produced from segmentation. Segmented Result by all utilized techniques are shown in Table 1.

**TABLE 1:** Comparison of computational time and object segmentation accuracy.

Datasets	Computational time			Segmentation accuracy (%)		
	DBSCAN	FCM	MRF	DBSCAN	FCM	MRF
UCM	162.13s	165.10s	**148.13s**	86.65	89.32	**91.18**
AID	175.30s	140.15s	**142.25s**	89.50	90.43	**91.67**

DBSCAN, Density Based Cpatial Clustering; FCM, Fuzzy Cmean; MRF, Markov Random Field; UCM, UC Merced; AID, Aerial Image Dataset. Bold values indicates proposed results (highlighed).

#### 3.3.1 Fuzzy C-mean segmentation

**FIGURE 3:**
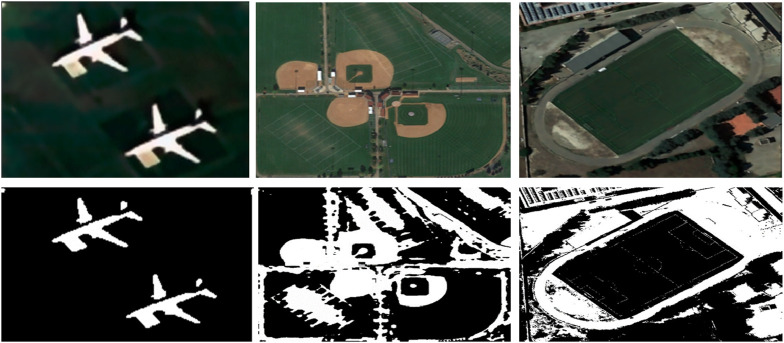
Fuzzy C-mean segmentation over some images from the UCM Dataset (row 1) represents the filtered images (row 2) demonstrates the segmented images.

This section explains the Fuzzy C-Mean (FCM) segmentation process. It begins by using pixels as data points to detect similar components. The pixel is subsequently assigned to numerous clusters instead of just one using fuzzy logic (Zhou, et al., 2023), producing a fuzzy assignment. To get the desired result, the objective function in FCM is iteratively optimized (Naseer and Jalal, 2023). To deconstruct the image, this iterative procedure entails changing membership degrees and clustering centers (Chen, et al., 2018). Performance index *H*_*FCM*_ is formulated using Equation 4.

$$H_{FCM}=\left(Q,S\right)=\sum_{i=1}^{r}\sum_{b=1}^{N}z_{ib}^{t}{∥q_{b}-s_{i}∥}^{2},1<\;t\;<\infty$$ (4)

where “*r*” is the set size of clusters, N is the size of pixels, *q*_*b*_ is the bth pixel, *s*_*i*_ is the ce nter of the ith cluster, and t is the blur exponent. Each cluster center and membership function are updated using Equations 5, 6.

$$z_{ib}^{t}=\frac{1}{\sum_{j=1}^{c}\left(\frac{1}{\left(t-1\right)}\right)}$$ (5)

$$s_{j}=\frac{\sum_{k=1}^{N}z_{ib}^{t}q_{b}\;}{\sum_{k=1}^{N}z_{ib}^{t}\;}$$ (6)

where $z_{ib}\in \left[0,1\right],for\;b=\left[1,\ldots ,c\right];\;l_{ib}^{2}$ represents the distance between pixel *q*_*b*_ and cluster centroid *s*_*i*_ and $z_{ib}^{t}$ stands for the membership matrix that belongs to [0, 1]. According to (Halder et al., 2011), FCM gives pixels close to the center of their class high membership values, whereas pixels far from the center receive low membership values. As demonstrated in Figure 3, which shows the segmented results of images from the UC Merced dataset, this processing complexity is applied to every nearby pixel in the images.

#### 3.3.2 DBSCAN clustering

**FIGURE 4:**
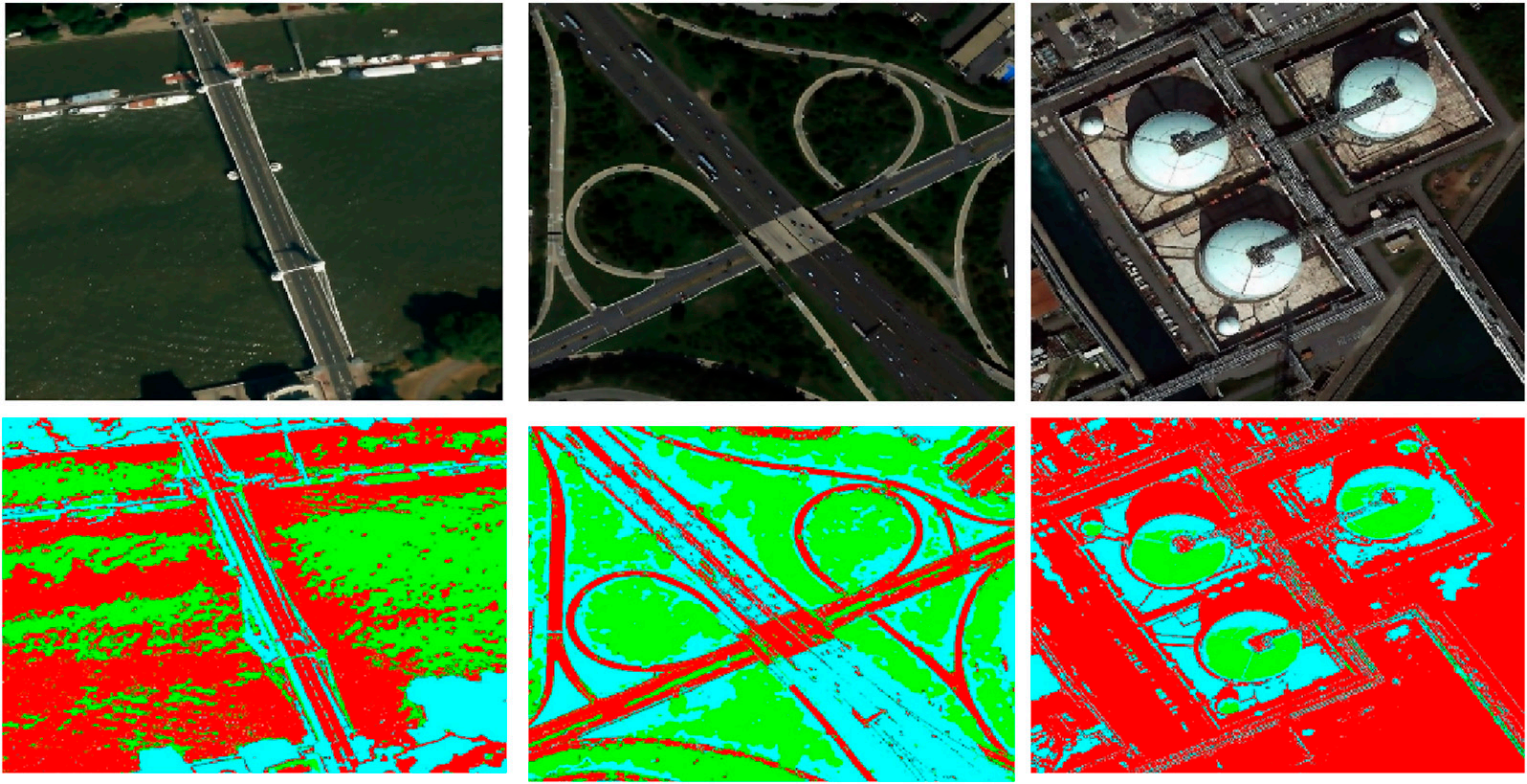
Density based clusters over some images from the AID Dataset (row 1) represents the filtered images (row 2) demonstrates the segmented images.

Density-Based Spatial Clustering or DBSCAN, is a popular clustering technique in the data analysis and machine learning domains. As opposed to conventional clustering techniques, which demand that the number of clusters be pre-specified, DBSCAN adopts a more data-driven methodology as in Equation 7. It is especially useful for discovering irregularly shaped and varying-sized clusters in complicated dataset since it clusters data points according to their density and closeness (Nawaz and Yan, 2020). The algorithm identifies core points as those with the fewest neighboring data points within a given distance Equation 8. After that, it adds neighboring data points that satisfy the density requirements, enlarging these core points into clusters (Jalal et al., 2021). Noise is defined as data points not fitting into any cluster or core point classification.

$$N_{\varepsilon }\left(x_{i}\right)=\left\{\;x_{j}\;\in X|dist\left(x_{i},x_{j}\right)\leq \varepsilon \right\},X=\left\{\;x_{1,}x_{2,\ldots ..}x_{n}\right\},$$ (7)

$$C=\left\{\;x_{i}\in X\|N_{\varepsilon }\left(x_{i}\right)\geq MinPts\right\}$$ (8)

where *x*_*i*_∈*X*‖*N*_*ε*_(*x*_*j*_) *and x*_*j*_∈*C*; *x*_*i*_ is the epsilon neighborhood of *x*_*j*_ and *x*_*j*_ is the core point. Figure 4 representing the outcomes in which density based clusters are form.

#### 3.3.3 Markov Random Field (MRF)

**FIGURE 5:**
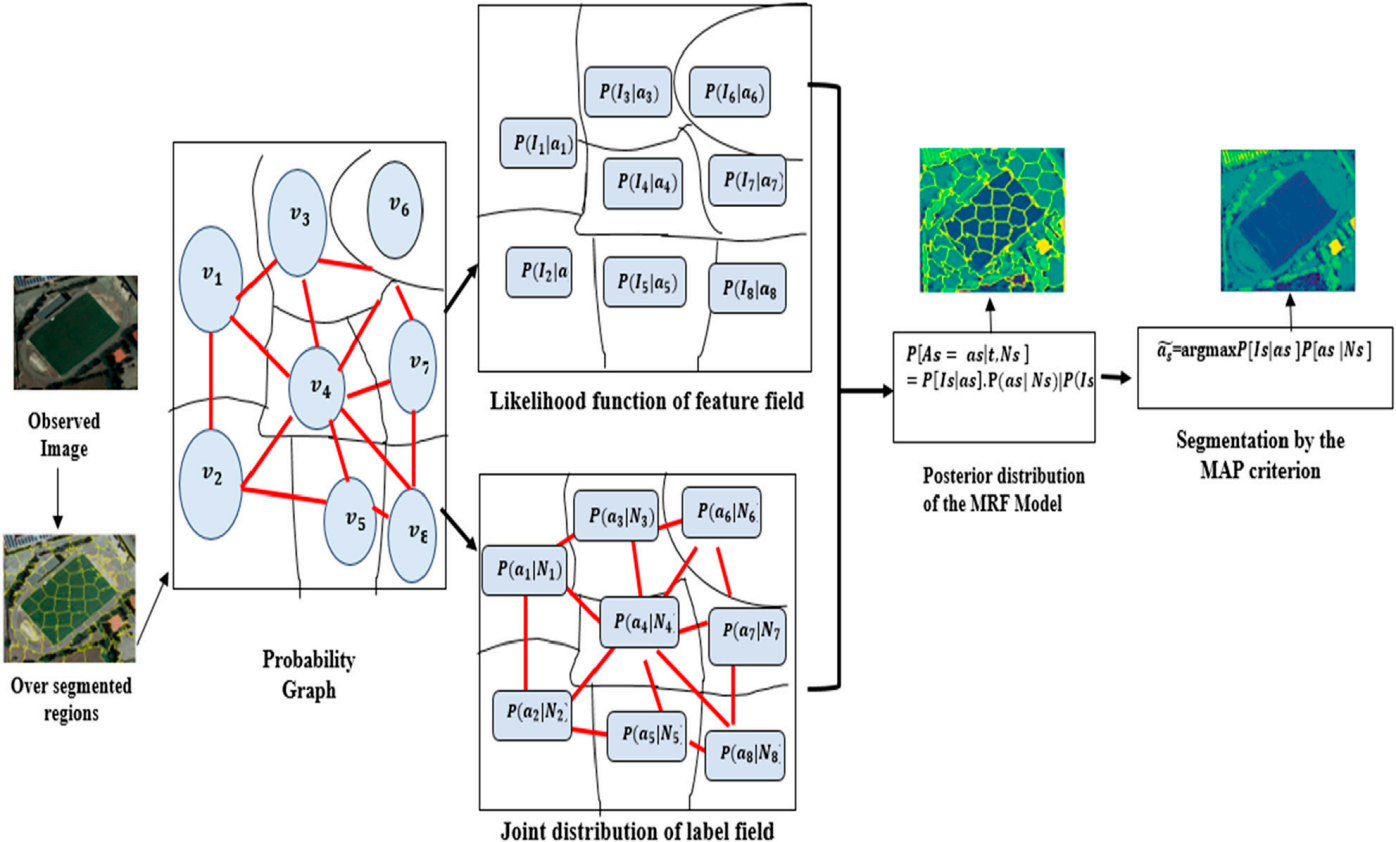
Illustration of the MRF model over image segmentation.

Consider G = v, e be the Markov Random Field (MRF) model’s probabilistic network (Zheng et al., 2019) The vertex collection is represented by v = {*v*_*s*_ | s ∈ S}, while the edge set is written as E = {*e*_*s*,*t*_ | s, t ∈ S}. In the probabilistic graph, a single site is denoted by “s”, while the whole collection of these sites is repesented by “S”. *e*_*s*,*t*_ = 1 if vs and vt are next to one other in space; *e*_*s*,*t*_ = 0 otherwise. In the traditional pixel-based MRF model, G is a probabilistic graph where each “s” denotes a pixel. In the MRF model, G is employed if “s” denotes an over-segmented region. Figure 5 shows the MRF model (Li et al., 2018), where *I*_*s*_ stands for the observed data of vs in the image I = {*I*_*s*_| s ∈ S} S stands for the label field *X* = [*Xs*|*s*∈*S*] in which the label class of each vs is represented by Xs, a random variable with values from Λ = {1, 2, …, k}. For instantiation of *A*,*a*= {*as*|*s*∈*S*}, the posterior probability *P*{*A* = *a*|*I*} may be found using Equation 9 on observed image I.

$$P\left(A|I\right)=\frac{P\left(I│A=a\right).P\left(A\right)}{P\left(I\right)}$$ (9)

where *P* (*A*|*I*) is the posterior probability, (*A*|*I*) is the likelihood of observing, P(A) is prior Probability and P(I) is the probability of observing I.

$$P\left(I|A\right)=\prod_{i=1}^{n}f\left(I_{i};A\right)$$ (10)

where *I*_*i*_ represents individual data points in the observed image (Lu et al., 2016). The spatial neighborhood interactions between labels of several places can be captured by the joint distribution as shown in Equations 11, 12 below.

$$P\left[Ac|At,t\in \frac{v}{vs}\;\right]=P\left[Ac|At,t\in Ns\;\right]$$ (11)

In this instance, if est = 1, then vt is in Ns, which means that Ns contains vs’s surrounding vertices. The Hammersley-Clifford theorem (Chen et al., 2017) uses potential functions to construct the joint probability distribution in MRFs.

$$P\left[\text{Ac}|\text{At},t\in \text{Ns }\right]=\frac{1}{Z\;}\prod_{c\in C}\varphi_{c\;}\left(A_{c}\right)$$ (12)

Z serves as the partition function to guarantee that the probabilities add up to 1, *A*_*c*_ stands for the variables in each clique, and C is the graph’s collection of maximal cliques. The possible function that relates to cliques are *φ*_*c*_, which represents the interaction between clique variables represented by c. According to the posterior prob. Of Equation 10. The segmentation of the image provided may be accomplished by finding the best realization by applying the MAP criterion Equation 13, i.e.,

$$\tilde{a}=argmaxP\left(A|I\right)=\mathrm{argmax}\left[P\left(I|A\right).P\left(A\right)\right]$$ (13)

Pair-site cliques are typically utilized to compute image segments *φ*_*c*_ (*A*_*c*_) in the *P* [*Ac*|*At*,*t*∈*Ns*] where *φ*_*c*_ (*A*_*c*_) = $\sum_{t\in N_{s}}V\left(a_{c},a_{t}\right)$ see Equations 14-16.

$$V\left(a_{c},a_{t}\right)=\left\{\begin{array}{c} -\vartheta ,a_{c}=a_{t}\\ \vartheta ,a_{c}\neq a_{t} \end{array}\right.$$ (14)

where ϑ is the potential parameter Thus enabling the representation of P (A= a):

$$P\left(A=a\right)=\Pi_{c\in C}\;P\left[A_{c}=a_{c},\left|t\right.,t\in Ns\;\right]=\Pi_{c\in C}\;\varphi_{c\;}\left(A_{c}\right)$$ (15)

**FIGURE 6:**
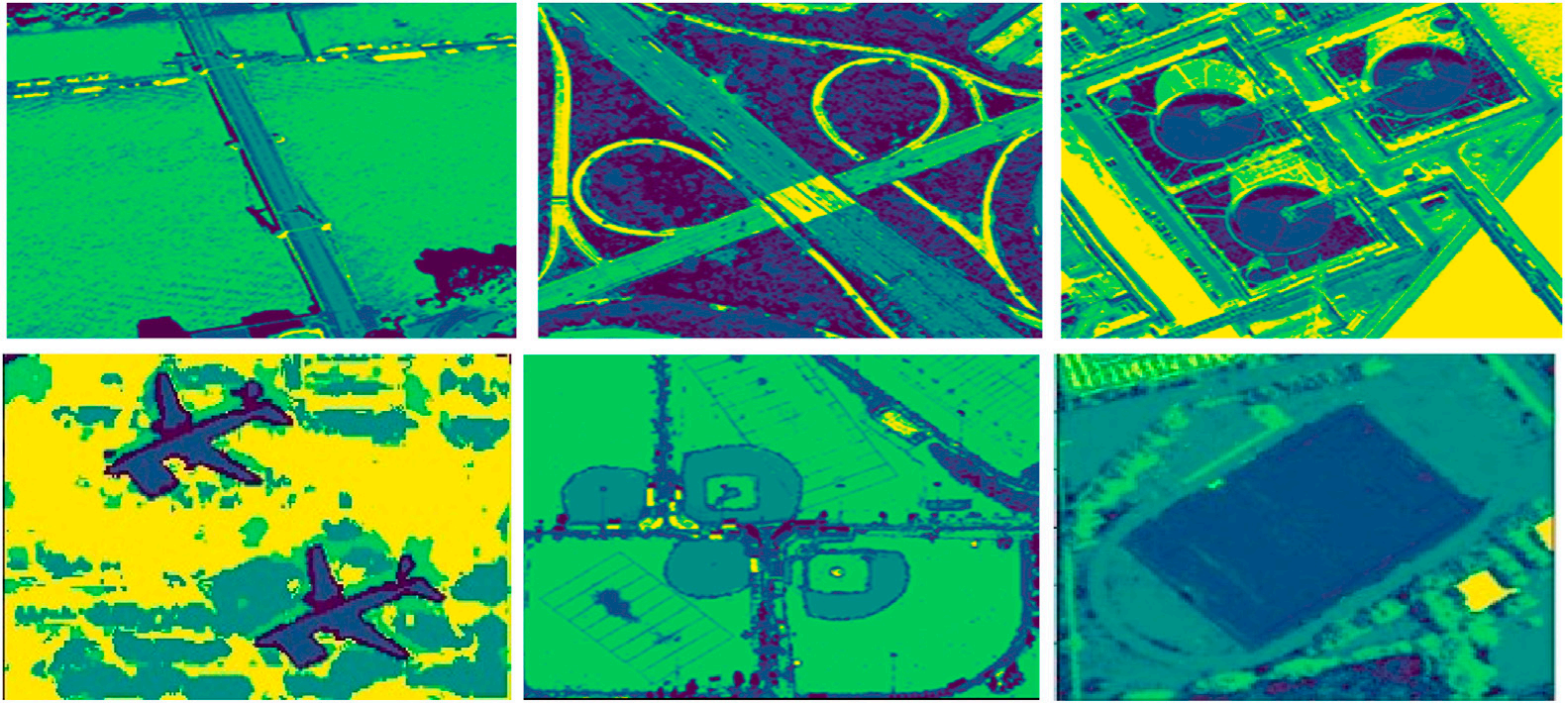
MRF-based segmentation over remote sensing images from both dataset (Row 1) AID dataset (Row 2) UMC dataset.

Therefore, by optimizing each $\tilde{{\;a}_{s}}$ , the optimal realization $\tilde{a}$ = { $\tilde{{\;a}_{s}}$ } may be attained progressively. The resultant segmentation obtained by using the MRF is shown in Figure 6.

$$\tilde{{\;a}_{s}}=argmaxP\left[A_{c}=a_{c}|I_{c},A_{t,}t\in \text{Ns}=\mathrm{argmax}\right]\left[P\left(I_{c}|A_{A}=a_{c}\right).P\left(A_{c}=a_{c}│A,t\in \text{Ns}\right)\right]$$ (16)

Based on the estimation time, three segmentation methods are contrasted. MRF-based segmentation is selected for labeling as it takes less time than DBSCAN and FCM. Labeling categorizes pixels by taking contextual information and nearby relationships into account. Using potentials or energies attached to pixel labels, MRF models these relationships (Nguyen et al., 2020).

### 3.4 Feature extraction

The extraction (Naseer and Jalal, 2023) of pertinent information or characteristics from aerial images is an essential phase in applications associated to remote sensing and image analysis. For object categorization in remote sensing images, a broad range of conventional features—including statistical techniques like texture, spatial, and spatial spectral features—are assessed. A thorough examination of the techniques for feature determination, combination, and selection is given in the following sections.

#### 3.4.1 Spatial features

**FIGURE 7:**
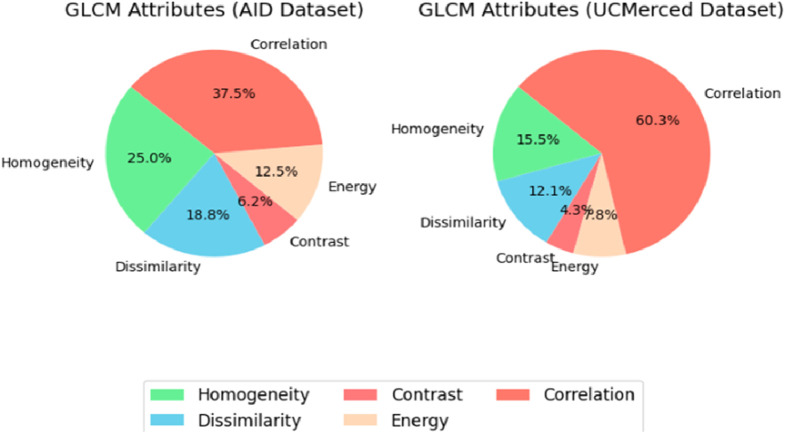
Distribution of GLCM attributes in AID and UC merced datasets.

The statistical measures known as Haralick texture characteristics are used to characterize the spatial arrangement or texture of pixel values in an image. The contrast, energy, entropy and correlation are calculated with the help of the gray-level co-occurrence matrix (GLCM). Using the GLCM (Sharma et al., 2016), we were able to extract textural properties and deduce four Haralick features shown in Figure 7. Based on the texture of the landscape, these characteristics—energy, correlation, contrast, and homogeneity—can be used to categorize different types of land cover given in Equations 17-20 respectively, such as urban and wooded areas. We utilized the texture data from the GLCM, which Haralick said included texture properties (Hema and Kannan, 2020) Specific formulae can be used to quantitatively compute the important texture properties.

$$Enr\left(I\right)=-\sum_{u=0}^{N-1}\sum_{v=0}^{N-1}{S\left(u,v\right)}^{2}$$ (17)

$$Corr\left(I\right)=\frac{\sum_{u=0}^{N-1}\sum_{v=0}^{N-1}\left(u,v\right)S\left(u,v\right)-\mu_{i}\mu_{j}}{\delta_{i}\delta_{j}}$$ (18)

$$Cont\left(I\right)=\sum_{u=0}^{N-1}\sum_{v=0}^{N-1}{\left(u-v\right)}^{2}P\left(u,v\right)$$ (19)

$$\text{HMG}\left(I\right)=‐\sum_{u=0}^{N‐1}\sum_{v=0}^{N‐1}P\left(u,v\right)P\left(u,v\right)$$ (20)

#### 3.4.2 spectral features

**FIGURE 8:**
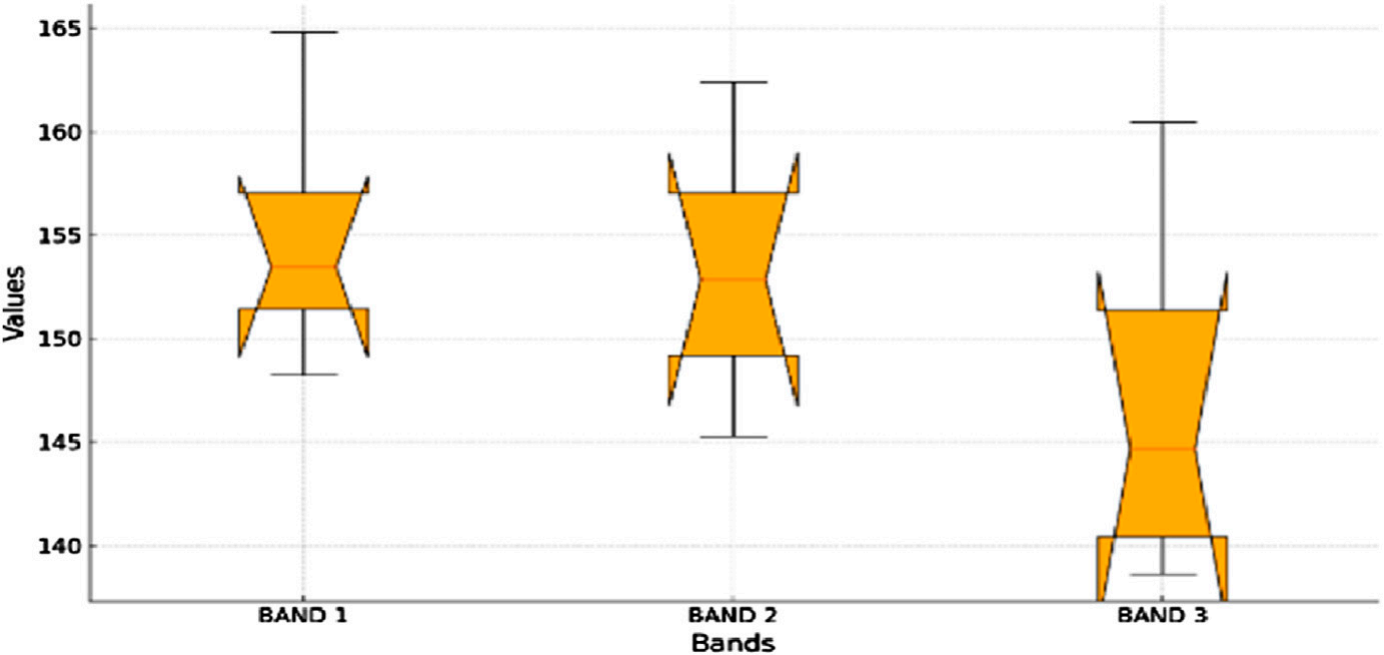
Box plot representing distribution of mean values across the different bands for all objects.

For Aerial imaging needs spectral features because they give important information about the makeup and characteristics of the Earth’s surface. Many spectral bands are frequently present in aerial shots, and provides distinct details about the electromagnetic energy reflected or emitted (Liang et al., 2018). These spectral properties are critical to many remote sensing and image processing applications. Spectral characteristics are mainly concerned with analyzing color information included in the image, which is usually obtained from many spectral bands. Metrics like the mean (Equation 21), standard deviation (Equation 22), and color histograms are examples of common spectral properties.

$$\bar{X}=\frac{1}{Z}\sum_{i=1}^{Z}x_{i}$$ (21)

$$\delta =\sqrt{\frac{1}{Z}{\sum_{i=1}^{Z}\left(x_{i}-\bar{X}\right)\;}^{2}}$$ (22)

where $\bar{X}$ is the mean, δ is the entire set of values in the spectral feature, Z is the entire set of values, and *x*_*i*_ is the individual value. Mean values are presented in bands in Figure 8, which indicates that medians for Bands 1 and 2 are relatively close, but somewhat lower in Band 3. It also shows that there is likely little overlap between the notches, or the confidence intervals and hence there is good evidence that the medians of the bands are significantly different. Moreover, it can be observed that Band 3 has a higher degree of variability than Bands 1 and 2, which can also be seen from the whiskers. These observations bring out different features of each band which is important when trying to solve the problem of identifying the features or objects in the dataset.

#### 3.4.3 Spatial spectral features

By separately computing different characteristics, such as textural features, spatial attributes, and spectral features, the approach yields Spectral Spatial Characteristics (SSFs), which are unique feature vectors. SSFs (Zhang et al., 2018) combine spatial and spectral (colour) data from representations of remote sensing. These elements include edge information, texture, spatial autocorrelation, and statistics derived from spectral bands (e.g., mean and standard deviation) that represent the spatial relationships among objects in the image together with their spectral qualities. SSFs are essential for distinguishing across groupings of land cover that have varied spatial layouts but comparable spectral features.

### 3.5 Feature fusion

In this experiment, we concatenate all the features to the feature vector. To make our dataset as useful as feasible, the objective was to find and keep only those characteristics that demonstrated substantial variance across all features. The Texture (GLCM), Spectral and SSF and are computed separately *Feature*_*GLCM*_, *Feature*_*SF*_ and *Feature*_*SSF*_ respectively. With reference to (Zhao and Du, 2016; Raja et al., 2020) a complete fused feature vector is created by the fusing of many feature vectors. Normalization prior to fusion is essential for balanced representation of features. It keeps a single element from taking center stage in the fused feature vector. Subsequent data processing is enhanced by this integrity preservation. The fused feature vector is composed of elements from the Haralick, Spatial, and SSF features combined together as shown below Equation 23.

$${Fused}_{F}=\left[\;{Feature}_{GLCM}\;{Featur}_{SF}\;{Feature}_{SSF}\right]$$ (23)

### 3.6 Dimensionality reduction using PCA

Dividing complex images into two layers produces a high-dimensional feature vector that can be used to manipulate the image. Working with high dimensions, meanwhile, can provide less-than-ideal outcomes. This problem is addressed by PCA feature selection for dimensionality reduction, which projects the data into a lower-dimensional space. This method, called Feature_RF, solves problems with high-dimensional data while optimizing processing time and computational resources.

In order to do this, the reduced dimensional feature vector *Feature*_*RF*_ is produced by using PCA feature selection as in Equation 24.

$${Feature}_{RF}=PCA\left[{Feature}_{F}\right]$$ (24)

**FIGURE 9:**
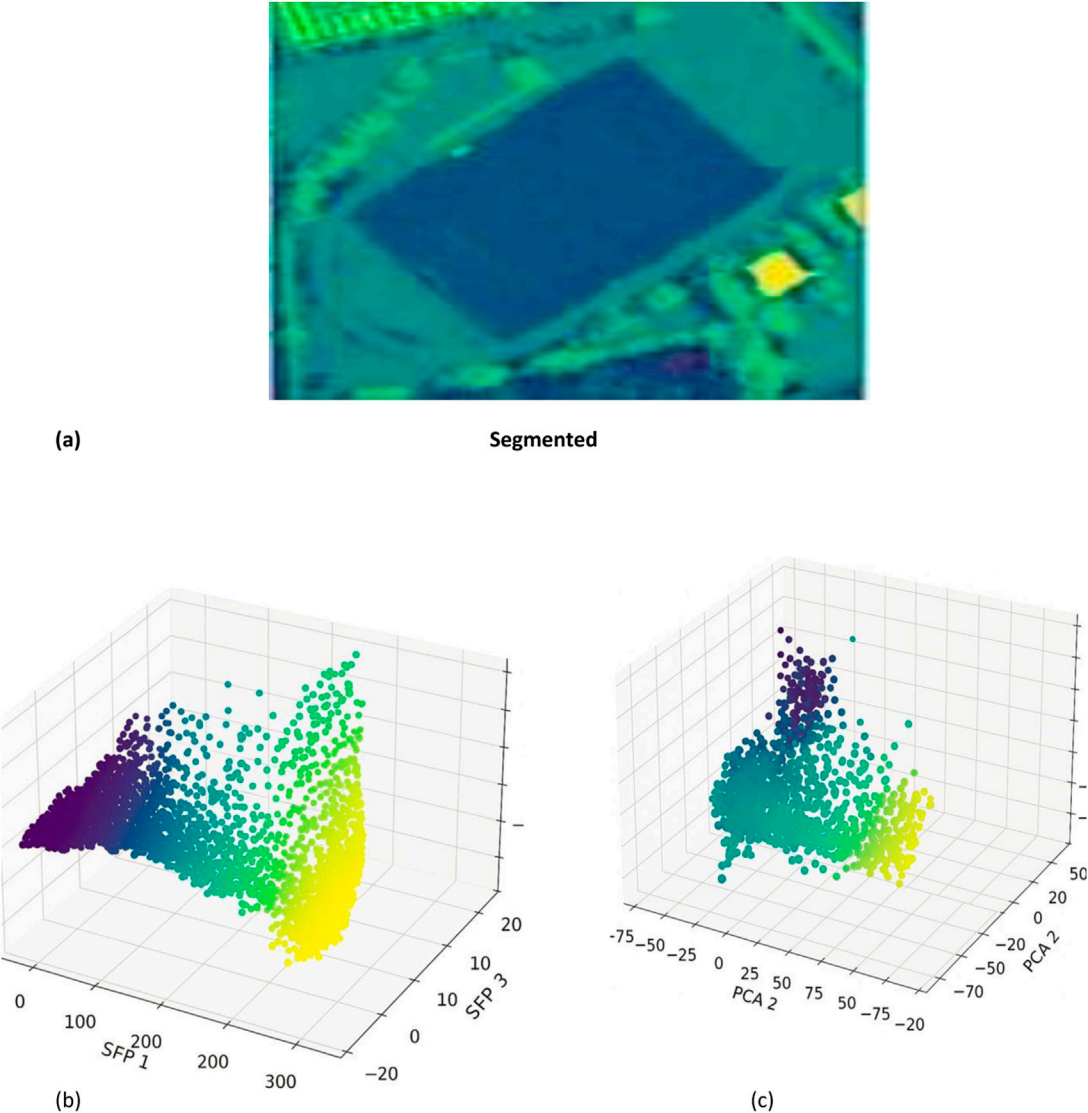
Dimensionality reduction **(a)** segmented **(b)** corresponding SSF extraction **(c)** PCA.

The SSF feature extraction of the combined features is shown in Figure 9, after which PCA is used to reduce dimensionality. The outcomes that followed are shown be.

### 3.7 Jaccard similarity

**FIGURE 10:**
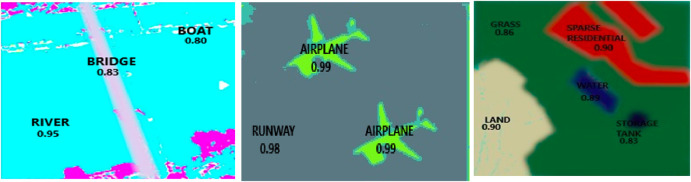
Jaccard jaccard similarity on multiple objects over some images.

The Jaccard Index, is a widely used metric to assess the degree of agreement between predicted masks and ground truth masks in pixel-level precision tasks like image segmentation. Figure 10 provides a visual representation of the segmentation algorithm achieves exceptional accuracy for large, distinct objects like “Airplane” (IoU: 0. While in larger and easier to identify objects such as “Bike” (IoU: 0.99) and “Runway” (IoU: 0.98), there is high accuracy, small and ambiguous object like “Boat” the same partially trained model yields an IoU of 0.80.

**FIGURE 11:**
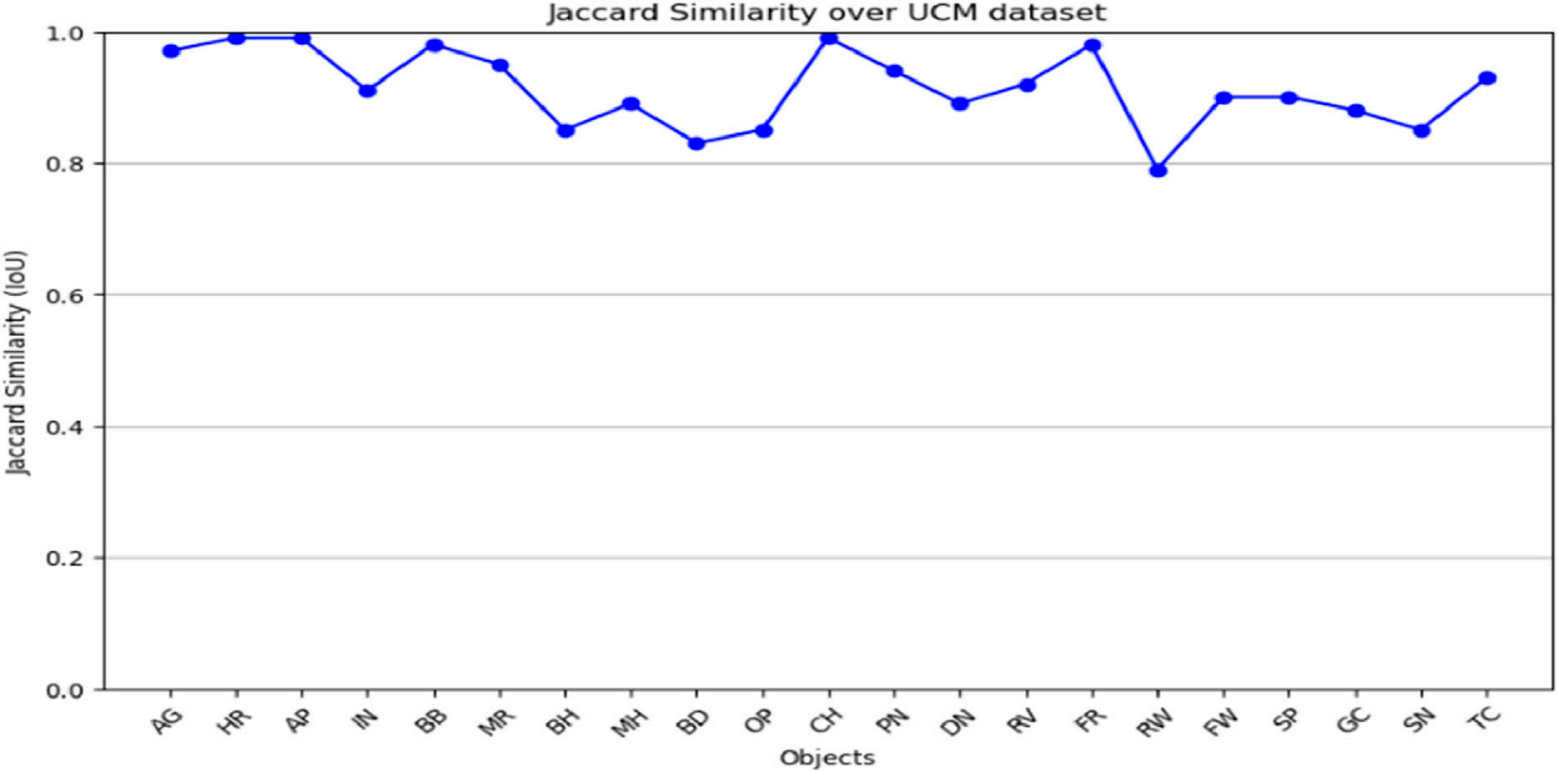
Jaccard similarity graph shown over UCM dataset.

**FIGURE 12:**
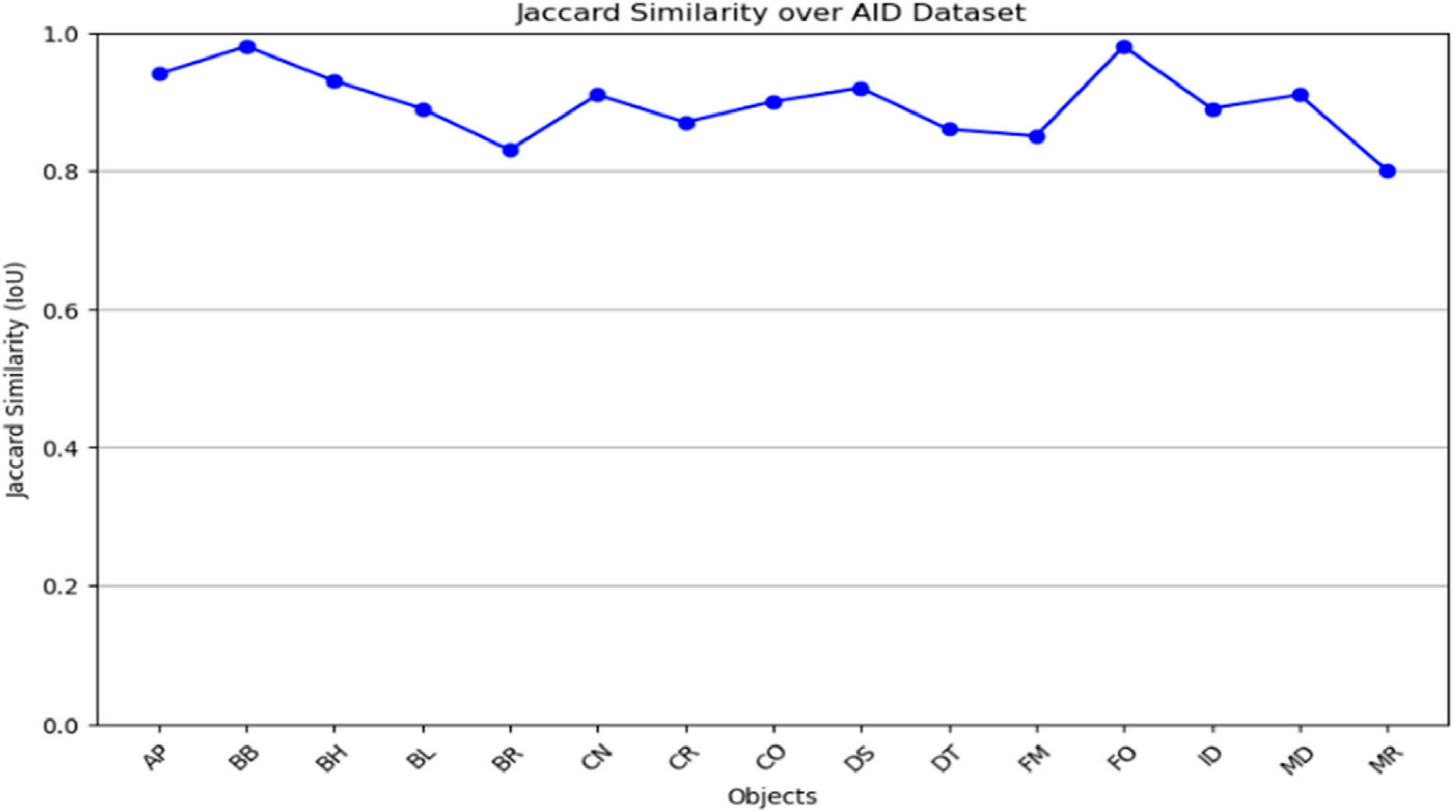
Jaccard similarity graph shown over AID dataset.

The graphs (Figure 11; Figure 12) evaluate segmentation quality across various object classes, highlighting both high-performing and challenging cases. The two graphs demonstrate the jaccard similarity (IoU) scores of objects classes in both AID and UCM datasets for an analysis of the segmentation performance of the different categories of objects. The segmentation algorithm works with reasonable accuracy based on the IoU scores following the delineated ranges: ≤0.5; 0.5–0.7; 0.7–0.8; and >0.8. In the case of both datasets, most of the IoU values are above 0.8. Out of all the classes in the AID dataset, we see near perfect IoU scores for certain classes like AP (Airport) and BB (Baseball Field) which are easily distinguishable under spectral-spatial characteristics. Still, the low values identifying such classes as MR (Meadow) indicate difficulties distinguishing between these objects because of their similarities with the surrounding environment. Likewise, in case of UCM dataset, classes like CH and AG are well detected where IoU value is close to one and FW and SP have relatively low IoU value. Altogether, these results demonstrate the effectiveness of the presented algorithm for most considered object classes with indication on the potential improvement of the algorithm’s performance in the most challenging circumstances.

### 3.8 Scene classification using AlexNet

**FIGURE 13:**
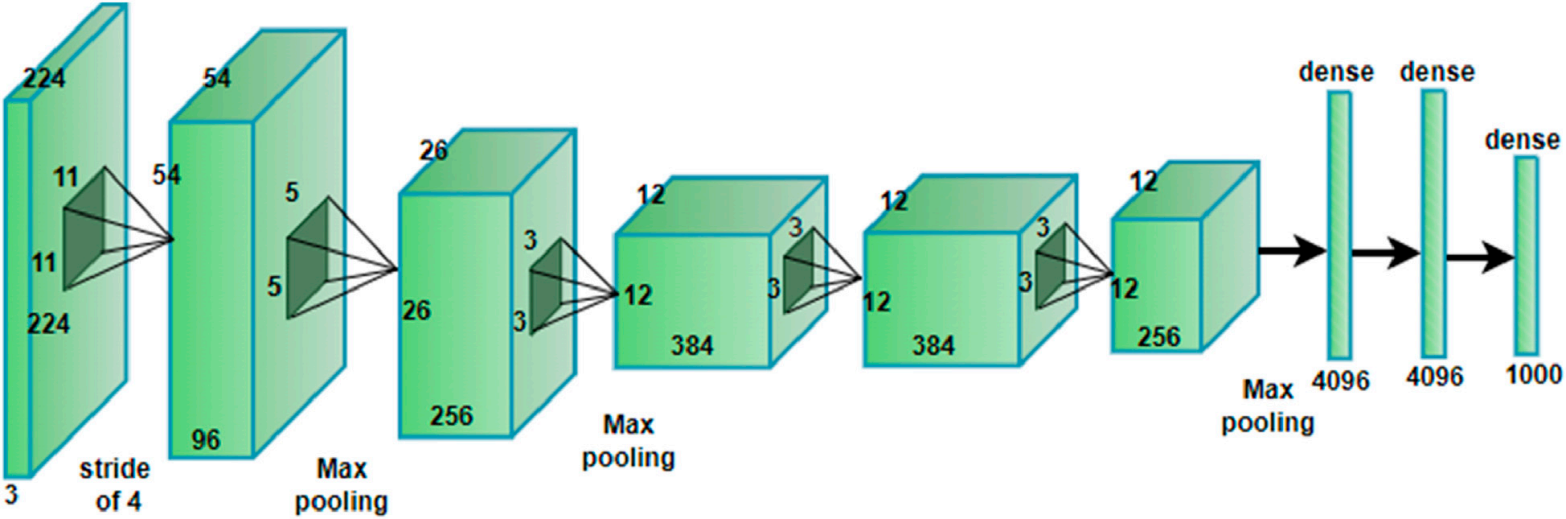
The layered architecture of the Alex Net.

The famous convolutional neural network (CNN) architecture known as AlexNet (shown in Figure 13) was created in 2012 by Alex Krizhevsky, Ilya Sutskever, and Geoffrey Hinton. It demonstrated exceptional performance in the ImageNet Large Scale Visual Recognition Challenge (ILSVRC) and was a major contributor to the deep learning for image classification field’s broad adoption.

This type of model takes an image input with dimensions of 224 × 224 × three and passing through several convolutions, pooling and fully connected layers to identify the input image and place it into one of the 1,000 categories. The First convolutional layer uses 96 filters of size 11 × 11 with four stride which down samples and aims to capture large scale features. Following layers’ use progressively smaller filters (5 × 5 and 3 × 3) with even more feature maps (up to 384) as in Equation 25 to identify the medium and even the fine scale features including the texture and edges. All these layers are said to follow some of the convolutional layers while they minimize the spatial dimensions while preserving the most important features in the images. Finally, following the final convolutional and pooling layers, there is a vectorization of the features maps, two dense layers containing 4,096 neurons each, to amalgamate the spatial features into a global space-quality representation. Last layer is the output layer that consists of 1,000 neurons, and performs softmax activation to drive out probabilities of the classes. Such a structure helps the model to learn higher levels of abstraction and for such tasks as object recognition the model is almost unbeatable.

$$Y=ReLU\left(convoluiton\left(X,W\right)+b\right)$$ (25)

The feature map (Y) is produced by convolution between (X, W), which is followed by the ReLU activation function. Spatial dimensions using Equations 26, 27 are then reduced by a max pooling layer using a 3 × 3 filter size and stride 2.

$$Y=\max\_pool\left(X,p\right)$$ (26)

**FIGURE 14:**
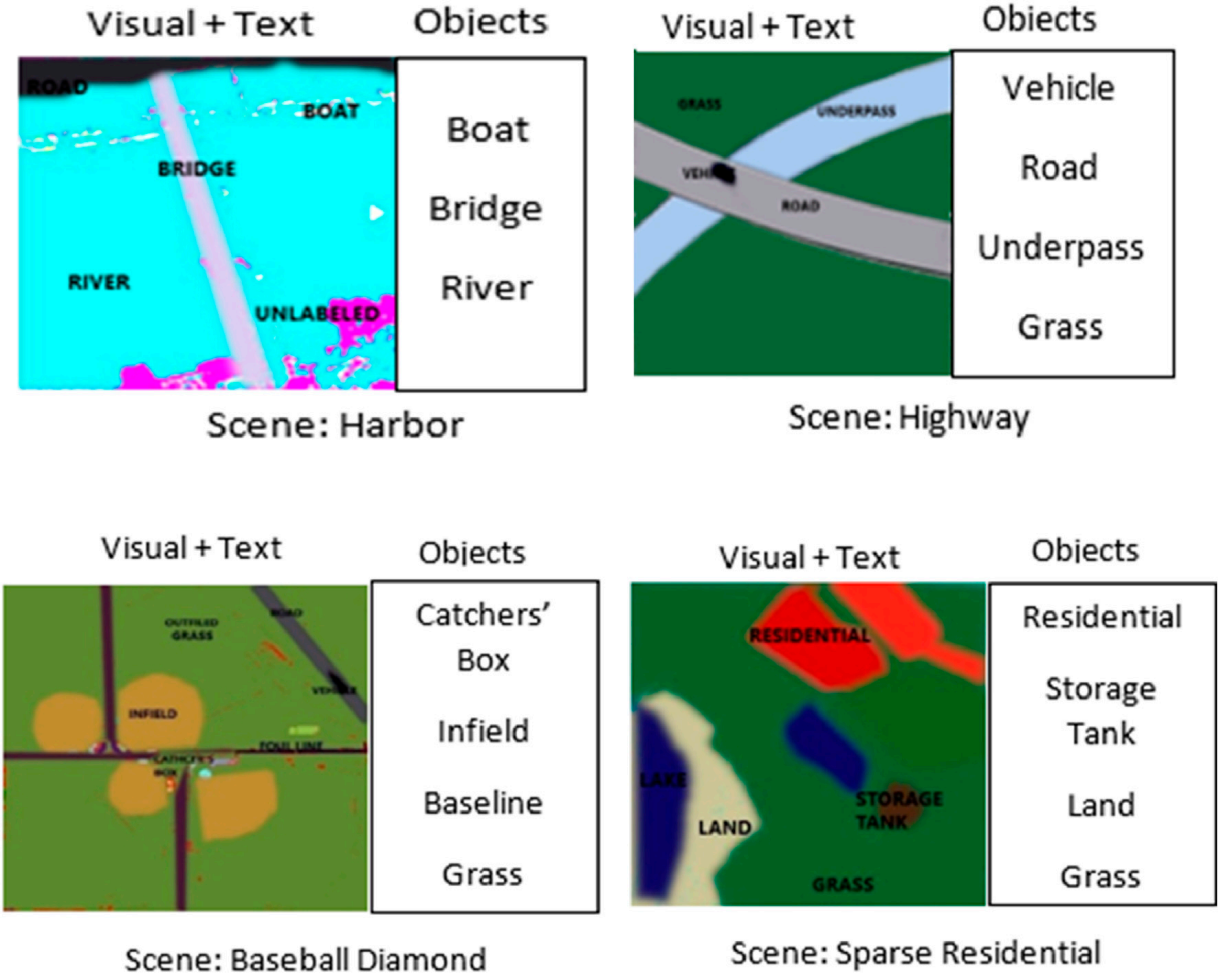
Scene recogntion using contextual relations between objects.

Yi,j,k=Xi,j,k/(k+α*∑Xi,j,kˋ^2^β (27)

where i, j, k representing spatial and depth dimensions, *k* ^*ˋ*^ represents the neighboring depth indices, *α* and *β* are hyper parameters. There are three fully connected levels in AlexNet. The first two layers each include 4,096 neurons, while the output layer contains 1,000 neurons, matching the classes in the UC Merced dataset. Softmax activation is used in the output layer to handle class probabilities. To keep the first two layers from overfitting, 0.5 probability dropout is applied to them during training. The usual training approach is stochastic gradient descent (SGD) with momentum (Thirumaladevi et al., 2023), and to increase the diversity of the training set, data augmentation techniques like random cropping and horizontal flipping are applied. Scene recognition has been done by the contextual relationship between multiple objects from remote sensing images using AlexNet shown in Figure 14.

In the presented framework, the reason for selecting AlexNet is based on its architectural effectiveness and its applicability to the requirements of Remote Sensing Image Analysis (RSIA) tasks. AlexNet’s hierarchical convolutional structure outperforms other deep architectures for detecting the small features at the initial layers such as edges and textures, and small semantic features at the later layers that are more relevant to scene recognition in complex data sets. This feature is considered a strength since it requires less computation and works efficiently on large datasets including the datasets AID and UCM it will not compromise performance with computational complexity.

Even though current progresses in architectures like ResNet or DenseNet provide deeper feature extraction in model architectures, they also bring more troubles like higher model complexity and stronger demand on computation resources which can an adversary against efficient implementations in certain situations. Moreover, the integration of AlexNet to the proposed Markov Random Field (MRF)-based segmentation enhances its performance to the maximum level, since it employs accurate and consistent segmentation outcomes for enhancing the feature learning process. The synergy that is tailored accordingly helped the system to obtain even more high classification rates 98.90% for UCM and 97.90% for AID databases, which is higher compared to many other state of the art methods. For that reason, AlexNet is poised to offer the best combination of efficiency, versatility, and reliability for this framework, and optimized for remote sensing use.

## 4 Experimental setup and dataset

Evaluation of the proposed system is performed on two benchmark datasets: Aerial Image dataset AID and UC Merced (UCM) dataset. The experiment is performed on an intel core i7 with 16 GB of RAM, a 3.2 GHz processor, and 512 GB of SSD.

### 4.1 Datasets description

#### 4.1.1 The aerial image dataset (AID)

The most current large collection of aerial images is called the Aerial Images Dataset (Xia et al., 2017). This dataset, which consists of 10,000 images overall across thirty classes of different scenarios. The collection includes a variety of aerial scene types, such as beaches, bridges, business districts, barren terrain, baseball fields, and airports.

#### 4.1.2 The UC merced dataset

A publicly accessible benchmark for study, the UCM dataset (Kim and Chi, 2021) consists of 100 images per class, all 256 × 256 pixels in size. These diverse images, which come from the USGS National Map Urban Area collection, include views of residences, beaches, farms, airplanes, and more.

### 4.2 Experimental evaluation

#### 4.2.1 Precision, recall, and F1-score

We provide recognition accuracies utilizing AlexNet and the OSCM architecture for the UCM and AID datasets. Our method uses ANN trained with SSF, Haralick, and spectral features, then XGBoost. Tables 2, 3 present a comparative evaluation of our OSCM framework and AlexNet for accurate scene recognition on difficult datasets utilizing Precision, Recall, and F1 Score measurements on the AID and UCM datasets.

**TABLE 2:** Results for scene recognition among three classifiers over AID dataset.

Categories	ANN			XGBoost			AlexNet		
	Pn	Rc	F1 Scr	Pn	Rc	F1 Scr	Pn	Rc	F1 Scr
AP	0.811	0.855	0.832	0.768	0.732	0.751	0.895	0.977	0.937
BB	0.871	0.917	0.893	0.883	0.857	0.869	0.960	0.955	0.957
BH	0.915	0.955	0.934	0.995	0.951	0.972	0.924	0.972	0.947
BL	0.903	0.845	0.873	0.986	0.937	0.960	0.977	0.911	0.945
BR	0.944	0.933	0.938	0.967	0.903	0.933	0.899	0.935	0.916
CN	0.887	0.841	0.865	0.844	0.875	0.850	0.844	0.889	0.865
CR	0.935	0.798	0.862	0.755	0.839	0.795	0.915	0.887	0.903
CO	0.868	0.899	0.895	0.872	0.921	0.895	0.872	0.954	0.911
DS	0.933	0.884	0.907	0.886	0.938	0.909	0.928	0.971	0.950
DT	0.887	0.815	0.850	0.985	0.954	0.969	0.971	0.892	0.969
FM	0.809	0.856	0.837	0.901	0.856	0.877	0.915	0.977	0.937
FO	0.845	0.881	0.862	0.883	0.965	0.922	0.811	0.892	0.922
ID	0.929	0.862	0.895	0.995	0.951	0.972	0.913	0.928	0.972
MD	0.899	0.918	0.902	0.798	0.937	0.936	0.886	0.966	0.925
MR	0.975	0.955	0.965	0.967	0.903	0.933	0.897	0.937	0.916
MN	0.798	0.912	0.859	0.844	0.889	0.879	0.912	0.901	0.906
PK	0.845	0.947	0.893	0.889	0.839	0.863	0.977	0.887	0.929
PN	0.811	0.886	0.846	0.872	0.921	0.895	0.900	0.946	0.954
PG	0.869	0.818	0.842	0.886	0.938	0.909	0.855	0.891	0.872
PD	0.905	0.875	0.842	0.985	0.954	0.969	0.925	0.917	0.921
PR	0.956	0.836	0.891	0.901	0.859	0.877	0.937	0.977	0.956
RT	0.854	0.825	0.840	0.883	0.965	0.922	0.871	0.871	0.871
RS	0.957	0.851	0.900	0.879	0.851	0.864	0.995	0.951	0.972
RV	0.991	0.888	0.936	0.986	0.937	0.960	0.956	0.879	0.915
SC	0.899	0.835	0.865	0.967	0.809	0.880	0.891	0.903	0.896
SP	0.879	0.925	0.899	0.844	0.855	0.850	0.819	0.916	0.864
SR	0.933	0.918	0.925	0.899	0.839	0.867	0.977	0.921	0.948
ST	0.789	0.857	0.821	0.872	0.875	0.873	0.887	0.911	0.898
SN	0.887	0.877	0.881	0.886	0.913	0.810	0.793	0.935	0.858
VT	0.895	0.798	0.843	0.845	0.866	0.800	0.985	0.905	0.943
Mean	**0.859**	**0.875**	**0.880**	**0.897**	**0.885**	**0.875**	**0.903**	**0.921**	**0.936**

^a^ AP, airplane; BB, baseball diamond; BH, beach; BL, bare land; BR, bridge; CN, center; CR, church; CO, commercial; DS, dense residential; DT, desert; FM, farmland; FO, forest; ID, industrial; MD, meadow; MR, medium residential; MN, mountain; PK, park; PN, Parking;PG, playground; PD, pond; PR, port; RT, railway station; RS, resort; RV, river; SC, school; SP, sparse residential; SR, square; ST, stadium; SN, storage tank; VD, viaduct; Pn, Precision, Rc, Recall. Bold values indicates proposed results (highlighed).

**TABLE 3:** Results for scene recognition among three classifiers over UCM dataset.

Categories	ANN			XGBoost				AlexNet	
	Pn	Rc	F1 Scr	Pn	Rc	F1 Scr	Pn	Rc	F1 Scr
AG	0.755	0.788	0.755	0.799	0.899	0.817	0.901	0.977	0.937
AP	0.711	0.744	0.875	0.815	0.875	0.844	0.883	0.965	0.922
BB	0.783	0.711	0.746	0.841	0.819	0.747	0.995	0.951	0.972
BH	0.792	0.658	0.737	0.844	0.889	0.844	0.986	0.937	0.960
BD	0.701	0.725	0.758	0.889	0.839	0.889	0.967	0.903	0.933
CH	0.745	0.715	0.875	0.872	0.921	0.872	0.977	0.839	0.903
DN	0.799	0.791	0.795	0.886	0.938	0.886	0.872	0.921	0.895
FR	0.783	0.711	0.746	0.985	0.954	0.985	0.886	0.938	0.911
FW	0.771	0.792	0.781	0.901	0.859	0.901	0.985	0.954	0.969
GC	0.730	0.717	0.961	0.883	0.965	0.883	0.925	0.917	0.969
HR	0.755	0.788	0.755	0.879	0.851	0.879	0.936	0.977	0.937
IN	0.711	0.744	0.875	0.986	0.937	0.986	0.871	0.871	0.922
MR	0.783	0.711	0.746	0.967	0.809	0.967	0.995	0.951	0.972
MH	0.792	0.658	0.737	0.845	0.856	0.850	0.956	0.879	0.96
OP	0.701	0.725	0.758	0.879	0.851	0.864	0.891	0.903	0.933
PN	0.874	0.845	0.859	0.986	0.937	0.960	0.819	0.916	0.859
RV	0.869	0.829	0.903	0.967	0.809	0.880	0.977	0.921	0.903
RW	0.872	0.851	0.895	0.844	0.855	0.850	0.887	0.911	0.895
SP	0.886	0.918	0.911	0.899	0.839	0.867	0.793	0.935	0.911
SN	0.965	0.934	0.969	0.872	0.875	0.873	0.799	0.916	0.895
TC	0.901	0.859	0.937	0.886	0.913	0.899	0.855	0.891	0.911
Mean	**0.893**	**0.881**	**0.922**	**0.922**	**0.911**	**0.915**	**0.923**	**0.915**	**0.946**

^a^ AG, agriculture; AP, airplane; BB, baseball diamond; BH, beach; BD, building; CH, chaparral; DN, dense residential; FR, forest; FW, Freeway GC, golf course; HR, harbor; IN, intersection; MR, medium residential; MH, mobile home park; OP, overpass; PN, parking; RV, river; RW, runway; Sp, Sparse Residential; SN, storage tank; TC, tennis court; ANN, Artificial Neural Network; XGBoost, eXtreme Gradient Bossting. Bold values indicates proposed results (highlighed).

#### 4.2.2 Second experiment: confusion matrix

**FIGURE 15:**
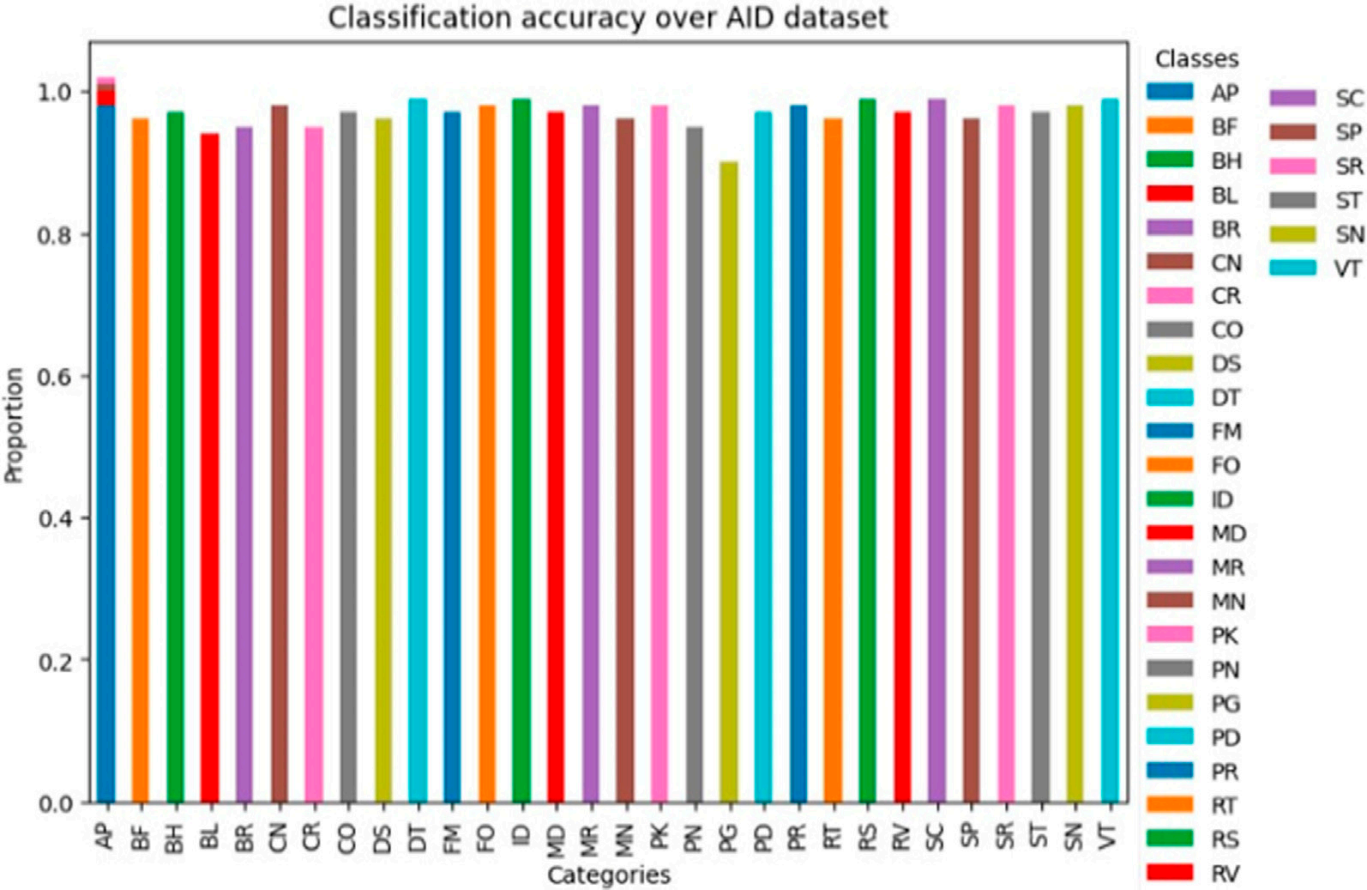
Scene classification accuracy over AID dataset.

**FIGURE 16:**
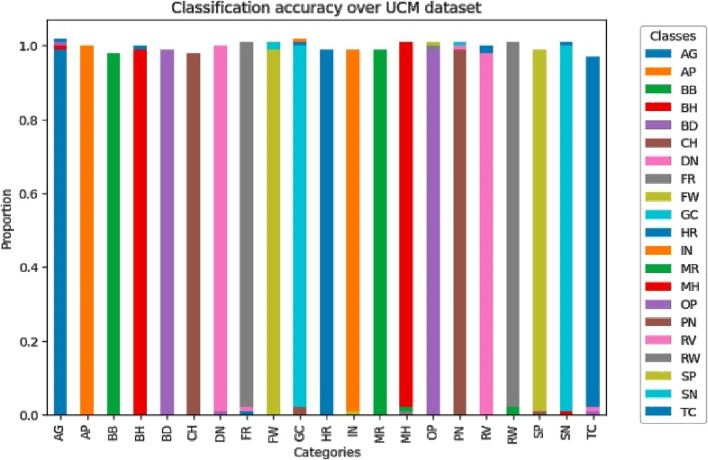
Scene classification accuracy over UC merced dataset.

**FIGURE 17:**
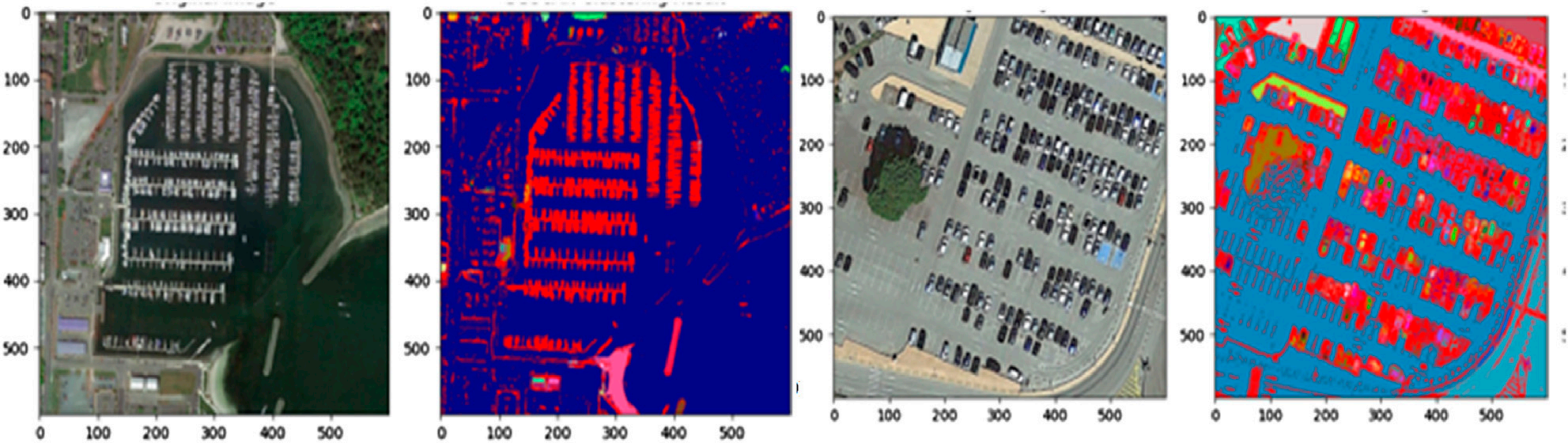
Failure cases where model does not perform well.

These two figures show how the classification rate varies on categories in AID and UCM sets. Every dataset shows a good level of performance of most classes with accuracy proportions being very close to 1.0% suggesting that all classes are well classified. Small deviations in some classes indicate where optimization is necessary more than ever, especially in classes that present a weak spectral and spatial contrast, as are observed within the Figures 15, 16 and failure cases shown in Figure 17.

We investigated comprehensively by contrasting our proposed approach with accepted state-of-the-art techniques. This evaluation was especially concerned with determining the average accuracy in object classification and segmentation. The results, presented in Table 4, provide a thorough comparison with the state-of-the-art methods now in use. These results show a significant improvement in performance, which we attribute to our novel OSC system.

**TABLE 4:** Comparison of the SOTA methods with the proposed OCS model.

Authors	Mean accuracies %	
	AID	UCM
Kim and Chi (2021)	86.91	86.79
Cheng et al. (2017)	—	94.17
Yu and Liu (2021)	—	84.00
Xie et al. (2021)	96.01	—
Wang et al. (2018)	88.75	96.81
Kollapudi et al. (2022)	—	90.29
Thirumaladevi et al. (2023)	—	95.00
Proposed	**97.90**	**98.90**

AID, Aerial Image Dataset; UCM, UC Merced Dataset. Bold values indicates proposed results (highlighed).

## 5 Ablation study

The proposed model, while achieving high accuracy and robustness, has certain limitations. First, its evaluation was limited to the AID and UC Merced datasets, which, although diverse, may not fully represent the complexity of real-world remote sensing scenarios. The model has not been tested on datasets containing multi-temporal or multi-sensor imagery, which could reveal its adaptability to varying data sources. Additionally, the lack of data augmentation techniques might have restricted the model’s ability to generalize further to unseen variations in the datasets. This will be done by a step-wise elimination or alteration of components, namely, the MRF segmentation, texture features based on Haralick, spectral-spatial features or particular layers of Alex Net and consequently measure the effect on performance. Such experiments will bring more attention to each module and will delete the unnecessary parameters improving the architecture. Ablation analysis shown in Table 5 will also allow for deeper understanding of how such hyper parameters affect model behavior to ensure that the level of accuracy and scalability is well understood.

**TABLE 5:** Ablation results for key features in the proposed model.

Experiment	Component removed	Accuracy (AID)	Accuracy (UCM)	F1 score (AID)	F1 score (UCM)
Full model	No	97.90%	98.90%	93.60%	94.16%
Without MRF	Replaced with FCM	92.50%	90.30%	92.10%	89.80%
Without spectral-spatial features	Removed	89.80%	87.60%	89.20%	86.90%
Without Haralick features	Removed	90.30%	88.20%	89.70%	87.50%
Without Bilateral Filter	Removed	85.60%	83.20%	85.10%	82.70%

## 6 Discussion and future work

The methodology proposed in this paper outperforms others through the use of MRF based segmentation, contextual feature extraction, and the recognition of scenes using Alex Net. Minimum segmentation accuracies of 91.18%, 91.67% of UCM, and AID datasets, respectively, further enhances the credibility of the proposed MRF model compared to DBSCAN and FCM. The addition of spatial, spectral and Haralick texture features aids in object discrimination whilst the incorporation of AlexNet aids in scene discrimination with accuracies of 97.90% on AID dataset and 98.90% on the UCM dataset. The novel method outperforms Xie et al. (96.01%) and Thirumaladevi et al. (95%) and establishes that the system can be successfully utilised for remote sensing. The generalizability of our proposed system to new circumstances is limited since it does not show how the OSC system works in real-world applications outside of the benchmark datasets.

Future research endeavors aimed at optimizing the OSC system’s efficacy will encompass the integration of advanced deep learning techniques, exploration of temporal and spatial transferability analysis, and evaluation of its robustness under diverse conditions. In addition, the model will be thoroughly examined by testing on several benchmark datasets and comparing its effectiveness with state-of-the-art technology.

## 7 Conclusion

The suggested OSC method performs exceptionally well at segmenting and categorizing objects in complex aerial data. We use a two-step strategy, where we use MRF for accurate segmentation and deep learning model Alex Net for scene recognition, using benchmark datasets. When processing complicated aerial images, our model performs better than state-of-the-art techniques, exhibiting outstanding accuracy and reliability. Our OSC system offers a dependable and complex solution with state-of-the-art methodologies and benchmark datasets, expanding the field of remote sensing research and creating new opportunities for aerial image analysis. In some images of classes (Parking, Port), our model does not perform well in distinguishing small and overlapping objects, accurately segmenting boundaries, and handling areas with similar textures, such as water and shadows. These limitations highlight the challenges in achieving precise segmentation in complex and densely packed regions.

## Data Availability

Publicly available datasets were analyzed in this study. This data can be found here: https://www.kaggle.com/datasets/abdulhasibuddin/uc-merced-land-use-dataset.
