# Correction: Multi-modal remote sensory learning for multi-objects over autonomous devices

**DOI:** 10.3389/fbioe.2025.1744642

**Published:** 2025-12-04

**Authors:** Aysha Naseer, Naif Almudawi, Hanan Aljuaid, Abdulwahab Alazeb, Yahay AlQahtani, Asaad Algarni, Ahmad Jalal, Hui Liu

**Affiliations:** 1 Department of Computer Science, Air University, Islamabad, Pakistan; 2 Department of Computer Science, College of Computer Science and Information System, Najran University, Najran, Saudi Arabia; 3 Computer Sciences Department, College of Computer and Information Sciences, Princess Nourah bint Abdulrahman University (PNU), Riyadh, Saudi Arabia; 4 Department of Informatics and Computer Systems, King Khalid University, Abha, Saudi Arabia; 5 Department of Computer Sciences, Faculty of Computing and Information Technology, Northern Border University, Rafha, Saudi Arabia; 6 Department of Computer Science and Engineering, College of Informatics, Korea University, Seoul, Republic of Korea; 7 Guodian Nanjing Automation Co., Ltd., Nanjing, China; 8 Jiangsu Key Laboratory of Intelligent Medical Image Computing, School of Future Technology, Nanjing University of Information Science and Technology, Nanjing, China; 9 Cognitive Systems Lab, University of Bremen, Bremen, Germany

**Keywords:** multi-modal, remote sensing, multi-objects, autonomous devices deep learning, computer vision, posterior probability, likelihood estimation, scene analysis

In the published article, there was an error in **Affiliation 2** for authors Naif Almudawi and Abdulwahab Alazeb. Instead of “^2^Department of Information Systems, College of Computer and Information Sciences, Princess Nourah Bint Abdulrahman University, Riyadh, Saudi Arabia,” it should be “^2^Department of Computer Science, College of Computer Science and Information System, Najran University, Najran, Saudi Arabia.”

In the published article, there was an error in **Affiliation 3** for author Hanan Aljuaid. Instead of “^3^Department of Computer Science, College of Computer Science and Information System, Najran University, Najran, Saudi Arabia,” it should be “^3^Computer Sciences Department, College of Computer and Information Sciences, Princess Nourah bint Abdulrahman University (PNU), Riyadh, Saudi Arabia.”

The original version of this article has been updated.

